# Adoption of an infection prevention and control programme (IPCP) in the Republic of Kiribati: a case study in diffusion of innovations theory

**DOI:** 10.1186/1753-6561-5-S6-O20

**Published:** 2011-06-29

**Authors:** P-A Zimmerman, H Yeatman, M Jones, H Murdoch

**Affiliations:** 1 Faculty of Health and Behavioural Sciences, University of Wollongong, Wollongong, Australia; 2 Faculty of Commerce, University of Wollongong, Wollongong, Australia; 3 Infection Control/Nursing Services, Ministry of Health, Tarawa, Kiribati

## Introduction / objectives

This paper presents a study which holistically examined the innovation processes experienced by the Republic of Kiribati in their adoption of a comprehensive IPCP innovation package.

## Methods

A case study methodology was used to explore IPCP adoption. Data sources and analysis included: 1) Chronological and thematic analysis of IPCP documentation and assessments performed by local staff and external agencies/consultants, and 2) semi-structured interviews with local key informants and external agencies (using snow-ball sampling) with thematic analysis. Analysis was informed by the Diffusion of Innovations for Organisations framework.

## Results

Identification of the two key activities of the innovation process for organisations, initiation and implementation (of the IPCP) was achieved. The initiation activity included two stages: 1) *agenda-setting:* preparations for severe acute respiratory syndrome (SARS) in 2003 stimulated the identification of organisational IPCP deficits, and 2) *matching:* IPCP deficits were identified and the decision to adopt an IPCP innovation package was made. Implementation included three stages: a) *redefining/restructuring:* identification of the components of an IPCP and how they best fit with the local health structure, b) *clarifying*: integration of IPCP into the health services and defining an infection control role within the nursing division and, c) *routinising:* the IPCP became an ongoing element in health service delivery.

## Conclusion

The adoption of the IPCP followed the classic Diffusion of Innovations Process for Organisations. This process can serve as an IPCP adoption model in other low- and middle-income healthcare settings.

## Disclosure of interest

None declared.

